# A decade of sustained geographic spread of HIV infections among women in Durban, South Africa

**DOI:** 10.1186/s12879-019-4080-6

**Published:** 2019-06-07

**Authors:** Gita Ramjee, Benn Sartorius, Natashia Morris, Handan Wand, Tarylee Reddy, Justin D. Yssel, Frank Tanser

**Affiliations:** 1 HIV Prevention Research Unit, South African Medical Research Council, 123 Jan Hofmeyr Road, Westville, Durban, KwaZulu-Natal 3630 South Africa; 2 Department of Epidemiology and Population Health, London School of Hygiene and Tropical Medicine, London, UK; 3 School of Medicine, Department of Global Health, University of Washington, Seattle, WA USA; 4 School of Nursing and Public Health, University of KwaZulu-Natal, Kwazulu-Natal, Durban, South Africa; 5 Biostatistics Unit: GIS, South African Medical Research Council, Durban, KwaZulu-Natal South Africa; 6 Kirby Institute, University of New South Wales, Kensington, NSW 2052 Australia; 7 Biostatistics Unit, South African Medical Research Council, Durban, KwaZulu-Natal South Africa; 8 Africa Health Research Institute, Durban, Kwazulu-Natal South Africa; 9 Research Department of Infection & Population Health, University College London, London, UK; 10 Centre for the AIDS Programme of Research in South Africa – CAPRISA, University of KwaZulu-Natal, Durban, Congella South Africa

**Keywords:** HIV, Spatial epidemiology, Mapping, Incidence, Risk factors, Heterogeneity

## Abstract

**Background:**

Fine scale geospatial analysis of HIV infection patterns can be used to facilitate geographically targeted interventions. Our objective was to use the geospatial technology to map age and time standardized HIV incidence rates over a period of 10 years to identify communities at high risk of HIV in the greater Durban area.

**Methods:**

HIV incidence rates from 7557 South African women enrolled in five community-based HIV prevention trials (2002–2012) were mapped using participant household global positioning system (GPS) coordinates. Age and period standardized HIV incidence rates were calculated for 43 recruitment clusters across greater Durban. Bayesian conditional autoregressive areal spatial regression (CAR) was used to identify significant patterns and clustering of new HIV infections in recruitment communities.

**Results:**

The total person-time in the cohort was 9093.93 years and 613 seroconversions were observed. The overall crude HIV incidence rate across all communities was 6·74 per 100PY (95% CI: 6·22–7·30). 95% of the clusters had HIV incidence rates greater than 3 per 100PY. The CAR analysis identified six communities with significantly high HIV incidence. Estimated relative risks for these clusters ranged from 1.34 to 1.70. Consistent with these results, age standardized HIV incidence rates were also highest in these clusters and estimated to be 10 or more per 100 PY.

Compared to women 35+ years old younger women were more likely to reside in the highest incidence areas (aOR: 1·51, 95% CI: 1·06–2·15; aOR: 1.59, 95% CI: 1·19–2·14 and aOR: 1·62, 95% CI: 1·2–2·18 for < 20, 20–24, 25–29 years old respectively). Partnership factors (2+ sex partners and being unmarried/not cohabiting) were also more common in the highest incidence clusters (aOR 1.48, 95% CI: 1.25–1.75 and aOR 1.54, 95% CI: 1.28–1.84 respectively).

**Conclusion:**

Fine geospatial analysis showed a continuous, unrelenting, hyper HIV epidemic in most of the greater Durban region with six communities characterised by particularly high levels of HIV incidence. The results motivate for comprehensive community-based HIV prevention approaches including expanded access to PrEP. In addition, a higher concentration of HIV related services is required in the highest risk communities to effectively reach the most vulnerable populations.

**Electronic supplementary material:**

The online version of this article (10.1186/s12879-019-4080-6) contains supplementary material, which is available to authorized users.

## Background

South Africa has over 7 million human immunodeficiency virus (HIV) infected individuals [1, 2]. HIV prevalence in the country varies greatly across the provinces [3] and is highest in KwaZulu-Natal (KZN). The reasons for the higher burden in KZN (particularly among young women and girls) are multi-faceted and dependent on complex economic and localised social, behavioural and cultural factors [4]. Coupled with high HIV infection rates, is the high prevalence and incidence of other curable sexually transmitted infections (STIs) [5]. Furthermore, factors such as age of sexual debut [6], having a male partner aged 25–34 [7, 8], being unmarried or not living with a partner [9], multiple sexual partners [10, 11], lack of condom use [1], living in a low antiretroviral therapy (ART) coverage area [12] and high mobility [13], have also been reported as contributing factors in a young woman’s vulnerability to the risk of HIV acquisition.

Given the persistent high HIV incidence rates, particularly among young women in KZN [14], it is clear that more effective approaches are required to tackle the epidemic [15–20]. One aspect receiving increasing interest is finer scale geo-spatial analysis of HIV infection patterns to facilitate more appropriate and geographically targeted interventions [21, 22]. Previous studies have frequently demonstrated the profound impact of HIV clustering on the spread of the disease [23–25]. Furthermore, given the current climate of declining funds and consistent with the global recommendations from the Joint United Nations program on HIV/AIDS (UNAIDS), optimal allocation of the limited resources requires identification of the geographical patterns and clustering of new HIV infections [26]. The HIV Prevention Research Unit (HPRU) of the South African Medical Research Council (SAMRC) had participated in several large-scale HIV prevention trials from 2002 to 2012. The trials have been conducted among several community-based research sites located across the greater Durban region. These trials primarily sought to determine the efficacy of various female initiated HIV prevention options; however, investigational products being tested found no effect on preventing HIV infections [15–19]. The primary objective of this study is to assess the geographical variations and clustering of HIV infections at a localized level in recruitment communities. Results from our study will guide policy makers to develop tailored prevention strategies in order to allocate scarce resources by targeting the most at risk individuals at a sub-geographical level.

## Methods

### Study area and population

**Fig. 1:**
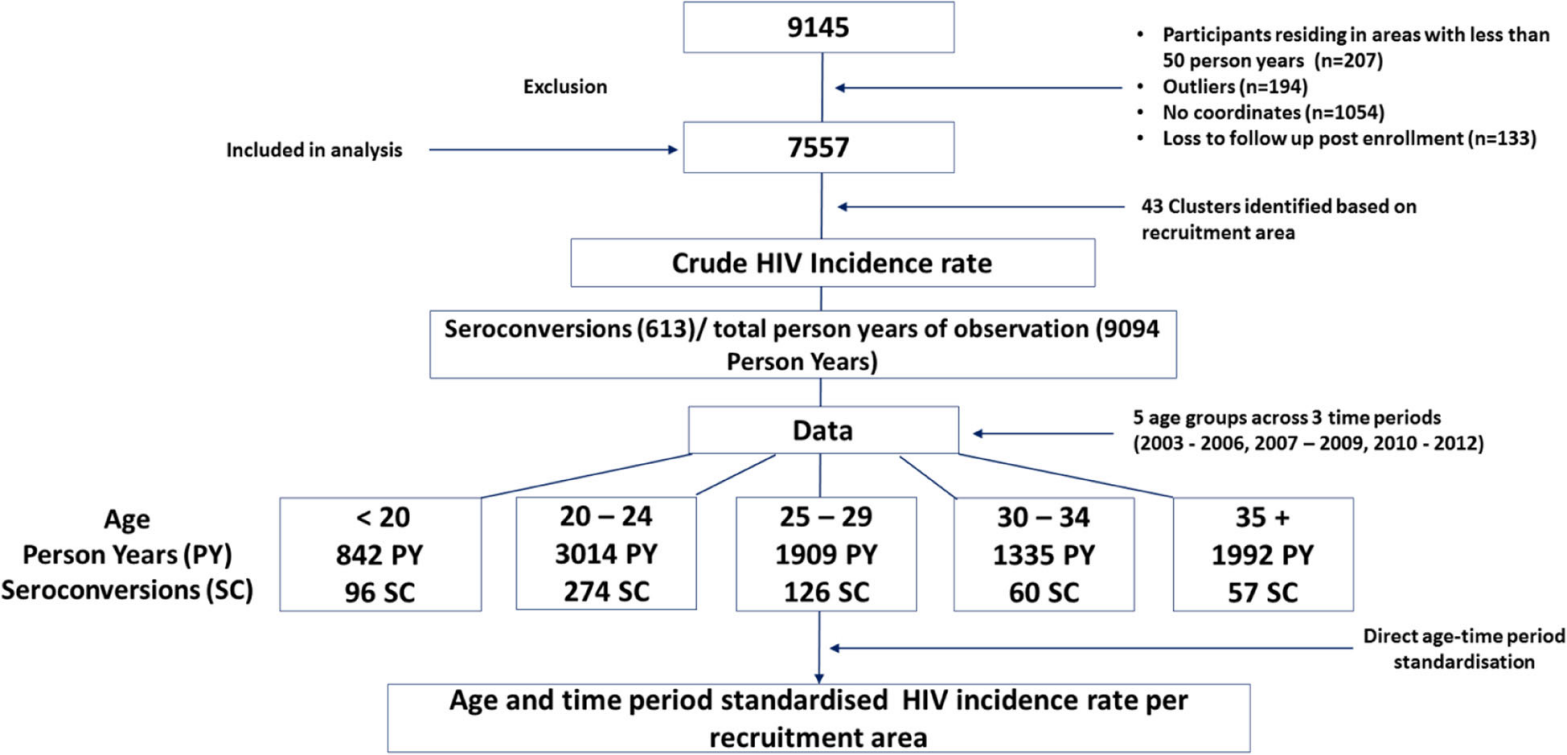
Flowchart for estimating age and time period standardized HIV Incidence rates

From 2002 to 2012, the HPRU of the SAMRC participated in five international multi-centre HIV prevention clinical trials [15–19]. A total of 9145 consenting women were enrolled in this combined cohort over the period of ten years. All consenting women’s places of residence (or nearest location point to residence) were geo-coordinated using Geographic Positioning System (GPS) at the time of enrolment and updated during follow-up visits (Additional file 2: Table S1 details the participating communities). We had access to GPS data of 7557 women, which were included in this analysis (Fig. 1).

GPS coordinate data were collected using Garmin™ Nüvi (model 2360) handheld devices, downloaded into Microsoft Access and plotted spatially using ArcGIS (version 10·4, CA). A total of 43 community-level recruitment area boundaries were developed based on census delineations [27].

Recruitment of trial participants was from communities in urban (peri-urban) and rural areas. Details of the study population have been described elsewhere [15–18]. For the purpose of the present analysis, data for women that were common across the clinical trials was extracted, combined and reported as unidentified and not study or site-specific [15–19, 28].

HIV incidence was the endpoint of all trials included in this analysis. HIV diagnostic testing was conducted using two rapid tests on whole blood sourced from either finger-prick or venepuncture (Determine HIV-1/2, Abbot Laboratories, Tokyo, Japan and Oraquick, Orasure Technologies, Bethlehem, PA, USA). The Abbot IMX Enzyme-linked immunosorbent assay (ELISA) test (Abbot Diagnostics, Africa Division), in combination with the Vironostika HIV1/2 ELISA was used on whole blood sourced from venepuncture for discordant/unequivocal results.

The age eligibility criteria were consistent across all trials (> 18 years of age) except for one which enrolled women aged 16 and older. Median age across the trials varied marginally between 24 and 28 years. The average screening to enrolment ratio was 47% in this combined cohort. Other eligibility criteria were broadly similar for all studies. At each visit, participants received HIV risk reduction counselling, STI testing and treatment and had access to male and/or female condoms. Women who tested HIV positive at screening were referred to local health care facilities for care and support. Women who HIV seroconverted during the trial remained in the study and received ongoing safe sex counselling, STI testing and treatment, and condom provision. All participants provided written informed consent to participate in the studies.

### Socio-demographic data collection

Data pertaining to age, contraceptive use, STI at screening (Chlamydia, Gonorrhoea, Syphilis and Trichomonas) and condom use at last sex act was consistent across all five clinical trials. Four of the clinical trials included marital status/cohabiting and parity data collection. The number of sex partners in the last three months was collected in three of the five clinical trials.

### Characterisation of high incidence communities

Participant data were classified into five age categories (< 20, 20–24, 25–29, 30–34, 35+ years old) across three-time ranges (2003–2006, 2007–2009 and 2010–2012). HIV incidence rates were calculated in each of fifteen strata (5 age groups and 3 time periods) in every recruitment area and a combined weighted estimate for each recruitment area was calculated. We employed direct age-time period standardization to obtain standardized HIV incidence estimates per area to facilitate legitimate geographical comparison (free from the influence of underlying differences in the age-composition of participants in different areas as well as overall incidence changes over time). The reference population was all women enrolled in the HIV prevention trials conducted at HPRU sites between 2002 and 2012.

The characteristics of the study population were compared across pre-defined HIV incidence rate categories (≤5, 5–6·9, 7–8·9 and 9+ per 100 person-year (PY)) as the dependent ordinal variable. This analysis utilised data at the individual women-level within this ordinal variable. Age, marriage/cohabitation, type of contraception used, STI at baseline, number of sexual partners in the last three months and parity data were included as the explanatory variables at the individual level. Univariable and multivariable ordered logistic regression, with recruitment area (community or group of communities) cluster robust standard errors, were used to identify prominent factors associated with higher incidence. Stepwise forward selection regression (inclusion if *p* < 0·10) was used to construct the final multivariable model. Adjusted odds ratios (aOR) and 95% confidence intervals (CI’s) are presented in Table 1. The proportional odds (or parallel regression) assumption for this modelling approach was checked and upheld (Brant’s test) [29].

**Table 1:** Characteristics of the study population by the categories of the HIV incidence rates and multivariable regression analysis

	Total N (%)	≤5 per 100PY	5–6.9 per 100PY	7–8.9 per 100PY	9+ per 100PY	Adjusted OR	*p*-value
Age group							
< 20	768 (10%)	75 (7%)	321 (11%)	199 (10%)	173 (10%)	1.51 (1.06–2.15)*	0.023
20–24	2606 (34%)	346 (33%)	986 (35%)	667 (34%)	607 (35%)	1.59 (1.19–2.14)*	0.002
25–29	1602 (21%)	239 (23%)	576 (21%)	382 (20%)	405 (23%)	1.62 (1.2–2.18)*	0.001
30–34	1027 (14%)	166 (16%)	386 (14%)	240 (12%)	235 (13%)	1.33 (0.98–1.82)	0.069
35+	1554 (21%)	231 (22%)	539 (19%)	443 (23%)	341 (19%)	1	
Education							
None	3322 (55%)	577 (56%)	1189 (52%)	637 (50%)	919 (60%)	1	
Primary	2485 (41%)	436 (42%)	992 (44%)	535 (42%)	522 (35%)	0.8 (0.7–0.92)*	0.002
Secondary or higher	281 (5%)	19 (2%)	89 (4%)	92 (7%)	81 (5%)	1.46 (1.1–1.95)*	0.009
Contraceptive use at baseline							
None	1079 (14%)	108 (10%)	416 (15%)	356 (18%)	199 (11%)	1	
Male/Female condom	1095 (15%)	125 (12%)	386 (14%)	305 (16%)	279 (16%)	1.14 (0.65–2.01)	0.639
IUD or Sterile	619 (8%)	107 (10%)	222 (8%)	183 (10%)	107 (6%)	1.28 (0.71–2.29)	0.406
Oral contraceptives	784 (10%)	168 (16%)	250 (9%)	146 (8%)	220 (13%)	1.71 (1.02–2.86)*	0.041
Injectables	3980 (53%)	549 (52%)	1534 (55%)	941 (49%)	956 (54%)	1.81 (1.1–2.96)*	0.019
Condom use at last sex							
Yes	4850 (66%)	704 (70%)	1859 (68%)	1079 (58%)	1208 (70%)		
No	2491 (34%)	303 (30%)	877 (32%)	792 (42%)	519 (30%)	1.09 (0.998–1.19)	0.056
Any STI							
No	6193 (82%)	900 (85%)	2293 (82%)	1569 (81%)	1431 (81%)	–	
Yes	1356 (18%)	156 (15%)	511 (18%)	361 (19%)	328 (19%)	1.04 (0.87–1.23)	0.664
Number of sex partners							
1	3929 (86%)	547 (89%)	1491 (86%)	1108 (85%)	783 (84%)	1	
2+	661 (14%)	69 (11%)	238 (14%)	199 (15%)	155 (16%)	1.48 (1.25–1.75)*	< 0.001
Parity							
0	757 (13%)	79 (10%)	342 (13%)	170 (11%)	166 (14%)	1.23 (0.91–1.68)	0.18
1	2610 (43%)	340 (42%)	1075 (43%)	634 (43%)	561 (46%)	1.13 (0.87–1.46)	0.356
2	1348 (22%)	212 (26%)	528 (21%)	326 (22%)	282 (23%)	1.09 (0.85–1.39)	0.489
3+	1317 (22%)	175 (22%)	575 (23%)	358 (24%)	209 (17%)		
Married/Cohabiting							
No	4467 (74%)	478 (59%)	1890 (75%)	1145 (77%)	954 (78%)	1.54 (1.28–1.84)*	< 0.001
Yes	1564 (26%)	327 (41%)	629 (25%)	344 (23%)	264 (22%)	1	

Note:

Age group, education, contraceptive use, number of sex partners and marital status were significant in mulivariable analysis. Adjusted odds ratios for condom use a last sex, STI at screening and parity were computed by adjusting for the aforementioned significant variables

Multivariable ordered logistic regression model based on participants who had complete data for all variables. The likelihood ratio test indicated that there was no violation of the proportional odds assumption in the final ordered logistic regression model that was fitted (*p*-value 0.100)

*aOR* Adjusted Odds Ratio

*IUD* Intrauterine device

*STI* Sexually-transmitted Infection

* Statistically significant (*p*-value < 0.05)

An aggregate of women’s residence per recruitment area representing a minimum of 50 PY were included in HIV incidence rate calculations (*n* = 7557) following exclusion of a) outlying participants not resident within core recruitment areas (*n* = 194), or residing in areas with less than 50 total PY (*n* = 207), (b) participants for whom no GPS data had been collected (*n* = 1054) and (c) participants who did not attend any follow up visits post enrolment (*n* = 133) (Fig. 1). The crude HIV incidence rate across all communities was calculated by dividing the number of seroconversions by total person-years of observation.

### Micro-geographical clustering analysis

We employed the most widely used Bayesian conditional autoregressive (CAR) hierarchical model to assess incidence risk across the 43 areas and mapped the relative risk for each community (Figure 3) [30]. Bayesian hierarchical models are one of the main statistical approaches for making inferences regarding the underlying relative risks of a given disease across often disjointed geographical areas. In addition to the parameters to account for shared boundaries (neighbouring areas) and unmeasured heterogeneity within areas, we also included additional covariate terms for age and time period to account for the potential confounding effect of these covariates and how they may vary across the 43 suburbs. We employed a Bayesian conditional autoregressive (CAR) hierarchical model to assess incidence risk across the 43 areas and mapped the relative risk for each community. Bayesian hierarchical models are most commonly used to address the problems posed by small area analysis. We utilised the Besag, York and Molliè (BYM) or convolution CAR model, which is formulated as follows:

$$ {O}_{i\sim } Poisson\left({E}_i{\gamma}_i\right) $$

$$ \log \left({\gamma}_i\right)=\alpha +{\varepsilon}_i+{\varphi}_i $$

where *γ*_*i*_ is the standardised relative risk in area i = 1 to 43, *O*_*i*_ is the number of incident HIV events in area i, *E*_*i*_ is the expected number of incidence events based on person time contribution, *ε*_*i*_ is the small area unstructured random effect term (to capture unstructured heterogeneity) and *φ*_*i*_ is the CAR spatial term (to capture structured variation). We used an adjacency matrix of common neighbouring areas (shared boundaries) of a given suburb when modelling this parameter whereby φ_i_ is the sum of the weighted neighbourhood values; ε_i_ was modelled assuming an independent normal distribution ${\varepsilon}_i\sim N\Big(0,{\sigma}_{\varepsilon}^2$) with variance ${\sigma}_{\varepsilon}^2$. Non-informative gamma priors were used for variance parameters in both the unstructured and structured random effects.

In addition to the classical parametrization of the model proposed by BYM we also included additional covariate terms for age and period of the individual within the regions 1 to 43 to account for the confounding effect of these covariates and how they may vary across the 43 suburbs. Markov chain Monte Carlo simulation (MCMC) was used to fit this model [30] and implemented in the Bayesian software package WinBUGS [31]. Visual inspection of the parameter series plots was used to assess model convergence as well as by using Gelman-Rubin statistics [32].

### Ethics approval and consent to participate

Ethical approval for the trials were received from the University of KwaZulu-Natal Biomedical Research Ethics Committee and the South African Medical Research Council ethics committee.

## Results

The total person-time in the cohort was 9093.93 years and 613 seroconversions were observed.

### Characterisation of the participants

Demographic characteristics and baseline sexual behaviour of women are presented overall and across the categories of HIV incidence rates (Table 1). The mean and standard deviation of age in the cohort was 27.75 and 7·86, respectively. More than 60% of the women participants were less than 30 years old. Majority of the women (> 80%) were unmarried and/or not living with their sexual partners and more than half of the women had no education. The most commonly used contraception at baseline were injectables (53%); while 10 and 15% of the study population reported using oral contraceptives and male/female condoms respectively. Approximately two thirds of women reported condom-use during their last sex act. At baseline, prevalence of STIs exceeded 18% in the 5–6.9, 7–8·9 and 9+ incidence categories compared with 15% in the ≤5 HIV incidence category. Most women had already given birth previously; specifically, 43, 22 and 22% reported having 1, 2 and 3 children respectively.

Compared to the women 35+ years old, younger women were significantly more likely to be in the highest HIV incidence category (aOR 1.51, 95% CI: 1.06–2.15; aOR 1.59, 95% CI: 1.19–2.14 and aOR 1.62, 95% CI: 1.2–2.18for < 20, 20–24, 25–29 years old respectively). Women who had two or more sex partners were 50% more likely to be in a higher HIV incidence area compared to women with a single partner (aOR 1.48, 95% CI:1.25–1.75; *p* < 0.001). A similar finding was observed for women who were unmarried or not cohabiting (aOR 1.54, 95% CI: 1.28–1.84; *p* < 0.001).

### Micro-geographical clustering analysis

**Fig. 2:**
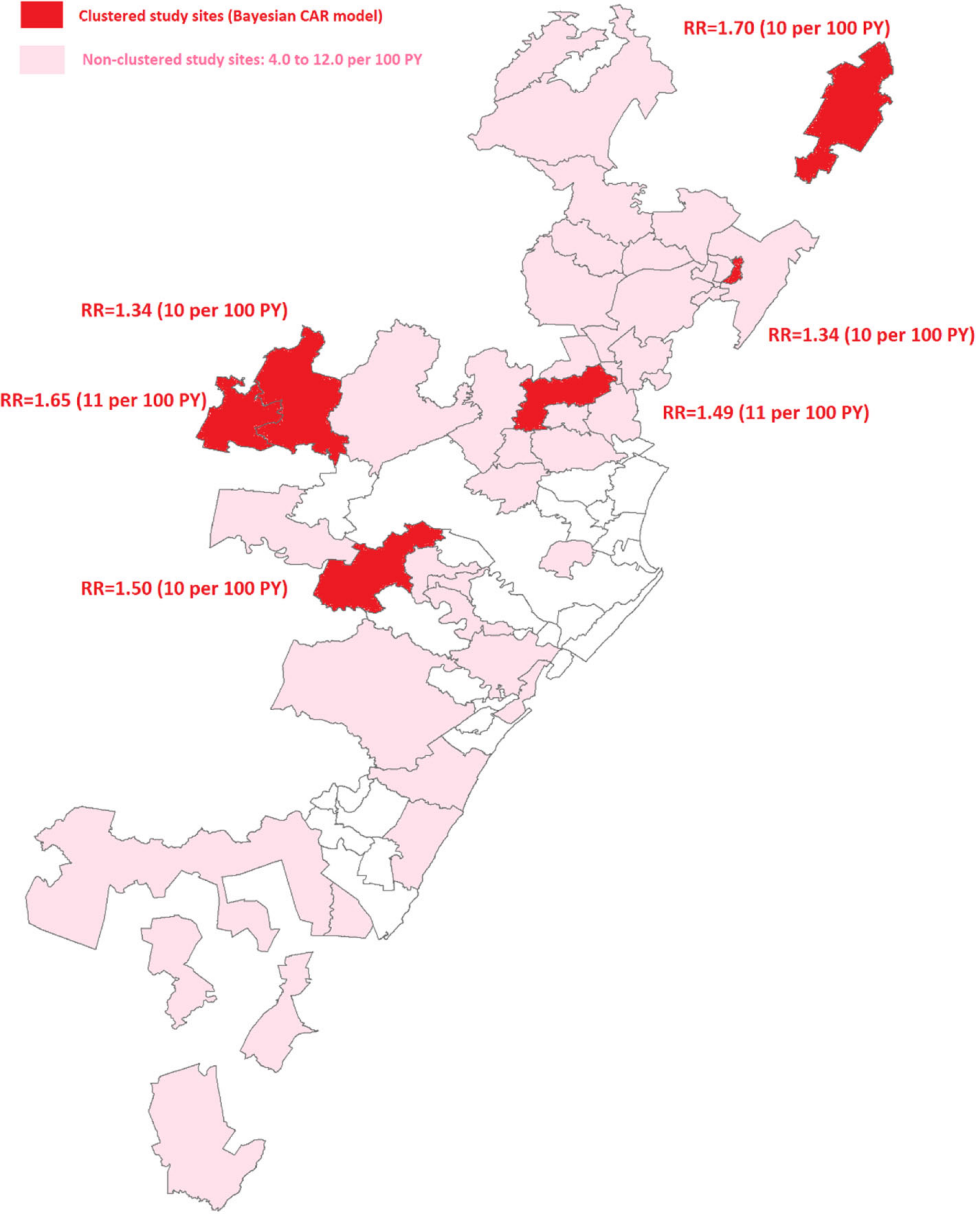
Relative risks (RRs) from the Bayesian conditional autoregressive (CAR) model and age-period adjusted HIV incidence rates (per 100 PY) for the six clusters with significantly higher incidence rate

Estimated relative risks (RRs) from the Bayesian CAR model were estimated across the 43 sub-geographical units and presented in Additional file 1: Figure S1. Our analysis identified six clusters located centrally and in the northern neighbouring areas of Durban (Figure 2). Estimated RRs for these clusters ranged from 1.34 to 1.70. Consistent with these results, age standardized HIV incidence rates were also highest in these clusters and estimated to be as high as 10 to 11 per 100 PY.

## Discussion

Through finer geospatial analysis of our data from over 7000 women participants, we observed a continuous and unrelenting hyper HIV epidemic in the greater Durban area over a ten-year period; with HIV incidence rates ranging from 3 to 12 per 100 woman-year (WY). These high HIV incidence rates were observed among trial participants who received regular HIV pre- and post- test counselling, safe sex counselling, treatment of curable STIs and male and female condom promotion. The high incidence rate observed during this period are similar to that reported from the CAP004 trial [33]; conducted between 2007 and 2009 in Durban. In that trial HIV incidence rates in the placebo group was reported to be 9/100 PY. Similarly, a cohort study by Nel et al. (2007–2009) observed an HIV incidence rate of 7.2/100 PY in the greater Durban area [34]. More recent data from the ASPIRE trial [35] showed that HIV incidence rates ranged from 5.4/100py to 8/100PY in the same communities from which this combined data was generated. The ASPIRE trial, suggested a slight decline in HIV incidence in these communities, However, rates as high as 8/100PY are unacceptable given the aggressive roll out of ARV treatment and access to standard prevention options in the country [35].

This is the first comprehensive study to analyse the geographic variation and clustering of HIV incidence rates among women in the greater Durban region. This study has several strengths which overcome many limitations which usually arise with averaged incidence maps (with often low number of events which make robust statistical interference difficult). We employed a Bayesian CAR model, which simultaneously estimates stable spatial and temporal structured patterns and departures from these stable components. In doing so we could capture spatial correlations or unobserved heterogeneity in HIV incidence that cannot be explained by time and other covariates. Results from this analysis underscored several pockets or clusters with high HIV infections in the greater Durban region. These areas were confined to central and northern territories. Previous work conducted in KwaZulu-Natal has examined the micro-geographical patterns of HIV prevalence [36] and HIV incidence in a rural setting over a decade of demographic surveillance [35]. However, no comparative work has been done in an urban setting in Northern KwaZulu-Natal. Similarly, our findings presented prominent heterogeneity in HIV rates in a relatively small geographic extent. In totality, these studies suggest apparent ‘corridors’ of elevated transmission [37] in the region which, based on the findings herein, may also be seen in other urban/peri-urban communities of KZN, as well as other similar hyper-endemic rural populations.

Concurrent with the previous studies, our results also provided additional evidence that these geographically clustered areas could be further differentiated with established risk factors for HIV seroconversion [6–12, 38–40]. As observed in our data, a large number of women, especially younger women, do not seem to be in a stable relationship and are likely to have multiple partners, use barrier methods less frequently thus potentially increasing their risk of HIV acquisition. This was further confirmed by high rates of other curable STIs. These findings are in keeping with those reported previously in KwaZulu-Natal [41]. Research on the role of STIs and inflammation and its impact on the vaginal microbiome and HIV acquisition risk is currently gaining international interest [42–44]. While behavioural and structural risks are important drivers of the HIV acquisition [45], considerable research is being undertaken to understand biological risk of HIV acquisition. It is more likely that in our region, all three risks play a significant role in the risk of HIV acquisition. Consistent with the previous studies, we also showed that women who use injectable and oral contraceptives are at a greater risk of being in a high HIV incidence category. These findings are similar to what we observed in another study [46].

Our results and that of others [41] have consistently shown that younger women are at a greater increased risk of being in the higher HIV incidence categories [47]. More recently, Akullian et al. [8] and de Oliviera et al. [7] showed that when these young women form sexual relationships with men aged 25–34 years of age, they are at particularly high risk for acquiring HIV.

South Africa has the largest ART programme in the world with just over 3.4 million individuals receiving medication [48]. The implementation of universal changes to national policies has been slow with sporadic uptake of ART from HIV positive participants, especially during the earlier period of the trials. This could explain, at least in part, some of the intra-community HIV incidence variability as well as the ubiquitously high rates of infection throughout. In addition, these findings provide further evidence for multiple sub-epidemics within a single region [49] in line with findings from KwaZulu-Natal [37] and other parts of Africa [50–52]. While understanding the spatial structure of HIV burden may allow for an appropriate concentration of services at a micro-geographic level, our data depicts an overall high HIV incidence among women in the entire greater Durban area and calls for a more blanketed and community-wide intervention and prevention efforts to have a major impact in this setting, with a particular focus on addressing the key drivers of the higher incidence identified among this population. In addition to this strategy, using the identified HIV clusters (that may be acting as a node for onward transmission) provides motivation for those communities to receive additional benefit from a more structured and concentrated HIV prevention and intervention approach. Targeting these HIV clusters may have numerous benefits. Firstly, such pockets of HIV are thought to re-seed nearby populations and may concurrently act as a potential “source of attraction” for HIV positive individuals from other communities. Secondly, targeting high risk areas is economically viable especially in resource constrained settings and provides an ideal starting point for ART implementation and scale-up of treatment as prevention (TasP); a strategy that is imperative to break onward HIV transmission. Indeed, without TasP implemented in such HIV concentrated pockets, one may observe a decreased efficacy of existing population-based intervention programmes. Furthermore, it has been previously demonstrated that ART adherence, and indeed uptake, is heavily negatively impacted by geographical barriers. It therefore makes placement of ARV clinics logistical, economical and ethical sense to target vulnerable individuals, aid adherence issues and ultimately achieve maximum penetration of HIV-related services to high-risk areas. This, in addition to a more blanketed intervention and prevention effort, may be the most successful strategy in reducing HIV incidence in a region with a burgeoning HIV epidemic.

The fact that socio-demographic information was not consistently collected across all trials, does pose a limitation and highlights the issue of missing data. A key issue in the extrapolation of data from clinical trials is the generalisability of the incidence data to women in the same geographical area. This may be a limitation of the study where we report on women, mostly of child-bearing age, that participated in a HIV prevention clinical trial. These women may perceive their risk for HIV as higher than the general population, due to individual circumstances and experiences. However, we utilised a consistent set of inclusion and exclusion criteria to enrol women across the decade of clinical trials. This consistency in eligibility (combined with the age and period standardisation approach employed) means that we are comparing a similar sample of women in all areas across time and space. It is thus unlikely that such selection effects could account for the remarkable spatial heterogeneity observed.

## Conclusion

Despite this unfortunate and rather unwelcome result, moving forward there is a clear need to intensify HIV treatment and prevention initiatives in a two-pronged approach. Firstly, due to the unacceptably high HIV incidence in all communities in Durban, an aggressive approach to combination prevention strategies including highly effective pre-exposure prophylaxis (PrEP) is needed. Although PrEP is registered for HIV prevention in the country, the roll-out programs are focussed on high risk groups such as men who have sex with men (MSM), sex workers and students. We make a case for universal roll out of PrEP in the greater Durban region with focus on individual risk assessment and provision and monitoring through innovative community-based programs. Secondly, those communities which are clear clustered transmission nodes, need a greater concentration of HIV related services (such as increased availability of HIV/STI clinics, access to treatment), where individuals at higher risk are targeted, and ultimately act as a powerful adjunct to prevention efforts to have a major impact in this setting.

## Additional files

Additional file 1: **Figure S1.** Community level HIV incidence rates using Bayesian conditional autoregressive (CAR) areal spatial regression analysis. (DOCX 313 kb)

Additional file 2: **Table S1.** Description of studies included in cluster analysis detection. (DOCX 14 kb)

## Abbreviations

aOR: Adjusted odds ratios
ART: Antiretroviral therapy
BYM: Besag, York and Molliè
CAR: Conditional autoregressive
CI: Confidence interval
ELISA: Enzyme-linked immunosorbent assay
HIV: Human immunodeficiency virus
HPRU: IV Prevention Research Unit
KZN: KwaZulu-Natal
MCMC: Markov chain Monte Carlo simulation
MSM: Men who have sex with men
PrEP: Pre-exposure prophylaxis
PY: Person-year
RRs: Relative risks
SAMRC: South African Medical Research Council
STI: Sexually transmitted infections
TasP: Treatment as prevention
UNAIDS: Joint United Nations program on HIV/AIDS
WY: Woman-year
