# Supplementary material for: A decade of sustained geographic spread of HIV infections among women in Durban, South Africa
Source: BMC Infect Dis. 2019 Jun 7;19:500. doi: 10.1186/s12879-019-4080-6 (PMC6555962; doi:10.1186/s12879-019-4080-6)
Supplement: Supplementary file 2 — Table S1. Description of studies included in cluster analysis detection. (DOCX 14 kb) [file 12879_2019_4080_MOESM2_ESM.docx]

**Supplementary Table**: Description of studies included in cluster analysis detection

| **Study name** | **Aim** | **Incidence rate**  **(greater Durban area sites only)** | **Reference (Main paper)** |
| --- | --- | --- | --- |
| Methods for Improving Reproductive Health in Africa (MIRA) | Tested the efficacy of diaphragm for HIV prevention | 6.7 per 100 PY | Padian NS, van der Straten A, Ramjee G, Chipato T, de Bruyn G, Blanchard K, et al. Diaphragm and lubricant gel for prevention of HIV acquisition in southern African women: a randomised controlled trial. Lancet 2007; 370:251–61. doi:10.1016/S0140-6736(07)60950-7. |
| Carraguard | A phase 3, randomized, placebo-controlled, double-blind trial, which was designed to investigate the efficacy and long-term safety of a carrageenan based gel formulation developed by the Population Council (New York, NY) for the prevention of HIV infection | 6.0 per 100 PY | Skoler-Karpoff S, Ramjee G, Ahmed K, et al. Efficacy of Carraguard for prevention of HIV infection in women in South Africa: a randomised, double-blind, placebo-controlled trial. The Lancet 2008; 372:1977-87. |
| Microbicides Development Programme (MDP)301 | A phase 3, randomised, double-blind, parallel-group trial investigated the efficacy of the PRO2000 vaginal gel | 7.1 100 PY | McCormack S, Ramjee G, Kamali A, Rees H, Crook AM, Gafos M, et al. PRO2000 vaginal gel for prevention of HIV-1 infection (Microbicides Development Programme 301): a phase 3, randomised, double-blind, parallel-group trial. Lancet 2010; 376:1329–37. |
| HIV Prevention Trial Network (HPTN) 035 | Evaluated the safety and effectiveness of two vaginal microbicides, Buffer Gel and PRO 2000/5, in preventing the transmission of HIV | 6.1 per 100 PY | Karim SSA, Richardson BA, Ramjee G, et al. Safety and effectiveness of BufferGel and 0.5% PRO2000 gel for the prevention of HIV infection in women. AIDS (London, England) **2011**; 25:957. |
| VOICE Trial | A randomized placebo-controlled trial that assessed the safety and effectiveness of oral tenofovir disoproxil fumarate (TDF), oral TDF plus emtricitabine (TDF-FTC), and vaginal 1% tenofovir (TFV) gel for HIV-1 prevention in women | 8.1 per 100 PY | Marrazzo JM, Ramjee G, Richardson BA, et al. Tenofovir-based preexposure prophylaxis for HIV infection among African women. New England Journal of Medicine **2015**; 372:509-18. |
