# Supplementary material for: A decade of sustained geographic spread of HIV infections among women in Durban, South Africa
Source: BMC Infect Dis. 2019 Jun 7;19:500. doi: 10.1186/s12879-019-4080-6 (PMC6555962; doi:10.1186/s12879-019-4080-6)
Supplement: Supplementary file 2 [file 12879_2019_4080_MOESM1_ESM.docx]

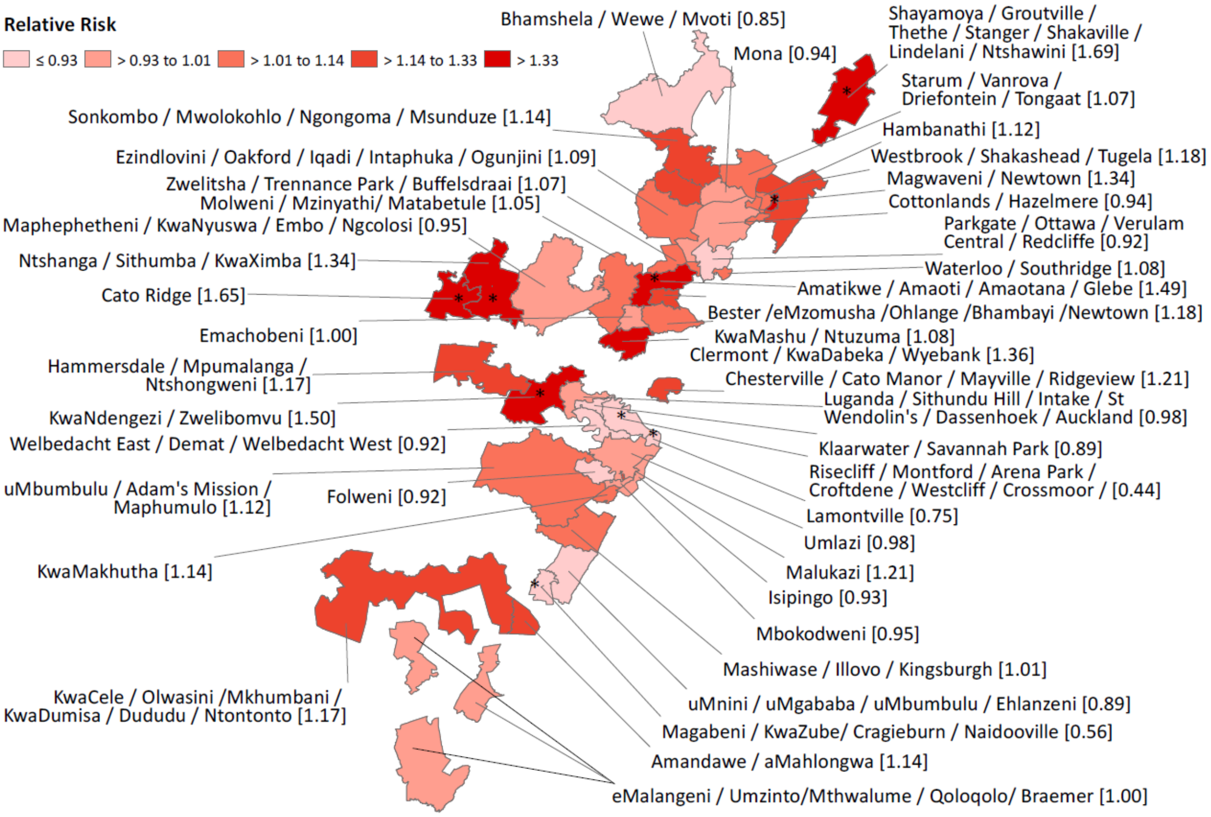

Relative Risk
≤ 0.93
> 0.93 to 1.01
> 1.01 to 1.14
> 1.14 to 1.33
> 1.33
Bhamshela / Wewe / Mvoti [0.85]
Shayamoya / Groutville / Thethe / Stanger / Shakaville / Lindelani / Ntshawini [1.69]
Mona [0.94]
Starum / Vanrova / Driefontein / Tongaat [1.07]
Sonkombo / Mwolokohlo / Ngongoma / Msunduze [1.14]
Hambanathi [1.12]
Ezindlovini / Oakford / Iqadi / Intaphuka / Ogunjini [1.09]
Westbrook / Shakashead / Tugela [1.18]
Zwelitsha / Trennance Park / Buffelsdraai [1.07]
Magwaveni / Newtown [1.34]
Molweni / Mzinyathi/ Matabetule [1.05]
Cottonlands / Hazelmere [0.94]
Maphephetheni / KwaNyuswa / Embo / Ngcolosi [0.95]
Parkgate / Ottawa / Verulam Central / Redcliffe [0.92]
Ntshanga / Sithumba / KwaXimba [1.34]
Waterloo / Southridge [1.08]
Cato Ridge [1.65]
Amatikwe / Amaoti / Amaotana / Glebe [1.49]
Bester /eMzomusha /Ohlange /Bhambayi /Newtown [1.18]
Emachobeni [1.00]
KwaMashu / Ntuzuma [1.08]
Clermont / KwaDabeka / Wyebank [1.36]
Hammersdale / Mpumalanga / Ntshongweni [1.17]
Chesterville / Cato Manor / Mayville / Ridgeview [1.21]
Luganda / Sithundu Hill / Intake / St Wendolin's / Dassenhoek / Auckland [0.98]
KwaNdengezi / Zwelibomvu [1.50]
Welbedacht East / Demat / Welbedacht West [0.92]
Klaarwater / Savannah Park [0.89]
Risecliff / Montford / Arena Park / Croftdene / Westcliff / Crossmoor / [0.44]
uMbumbulu / Adam's Mission / Maphumulo [1.12]
Folweni [0.92]
Lamontville [0.75]
Umlazi [0.98]
KwaMakhutha [1.14]
Malukazi [1.21]
Isipingo [0.93]
Mbokodweni [0.95]
Mashiwase / Illovo / Kingsburgh [1.01]
KwaCele / Olwasini /Mkhumbani / KwaDumisa / Dududu / Ntontonto [1.17]
uMnini / uMgababa / uMbumbulu / Ehlanzeni [0.89]
Magabeni / KwaZube/ Cragieburn / Naidooville [0.56]
Amandawe / aMahlongwa [1.14]
eMalangeni / Umzinto/Mthwalume / Qoloqolo/ Braemer [1.00]
